# Treatment- and Population-Dependent Activity Patterns of Behavioral and Expression QTLs

**DOI:** 10.1371/journal.pone.0031805

**Published:** 2012-02-16

**Authors:** Jesse D. Ziebarth, Melloni N. Cook, Xusheng Wang, Robert W. Williams, Lu Lu, Yan Cui

**Affiliations:** 1 Center for Integrative and Translational Genomics, University of Tennessee Health Science Center, Memphis, Tennessee, United States of America; 2 Department of Microbiology, Immunology and Biochemistry, University of Tennessee Health Science Center, Memphis, Tennessee, United States of America; 3 Department of Psychology, University of Memphis, Memphis, Tennessee, United States of America; 4 Department of Anatomy and Neurobiology, University of Tennessee Health Science Center, Memphis, Tennessee, United States of America; 5 Jiangsu Key Laboratory of Neuroregenertion, Nantong University, Nantong, China; Nagoya University, Japan

## Abstract

Genetic control of gene expression and higher-order phenotypes is almost invariably dependent on environment and experimental conditions. We use two families of recombinant inbred strains of mice (LXS and BXD) to study treatment- and genotype-dependent control of hippocampal gene expression and behavioral phenotypes. We analyzed responses to all combinations of two experimental perturbations, ethanol and restraint stress, in both families, allowing for comparisons across 8 combinations of treatment and population. We introduce the concept of *QTL activity patterns* to characterize how associations between genomic loci and traits vary across treatments. We identified several significant behavioral QTLs and many expression QTLs (eQTLs). The behavioral QTLs are highly dependent on treatment and population. We classified eQTLs into three groups: *cis*-eQTLs (expression variation that maps to within 5 Mb of the cognate gene), syntenic *trans*-eQTLs (the gene and the QTL are on the same chromosome but not within 5 Mb), and non-syntenic *trans*-eQTLs (the gene and the QTL are on different chromosomes). We found that most non-syntenic *trans*-eQTLs were treatment-specific whereas both classes of syntenic eQTLs were more conserved across treatments. We also found there was a correlation between regions along the genome enriched for eQTLs and SNPs that were conserved across the LXS and BXD families. Genes with eQTLs that co-localized with the behavioral QTLs and displayed similar QTL activity patterns were identified as potential candidate genes associated with the phenotypes, yielding identification of novel genes as well as genes that have been previously associated with responses to ethanol.

## Introduction

Understanding the genetic and molecular basis of natural variation in complex phenotypes has been a central goal of systems genetics [1]. Genome-wide association studies have been very successful in identifying genetic loci associated with phenotypes, and, in some cases, identifying differences in gene expression responsible for phenotype variation [2], [3], [4], [5]. However, genetic control of complex phenotypes has often been shown to be highly dependent on environmental and physiological conditions. Recently, several studies in yeast and mammalian cell lines [6], [7], [8] have attempted to exploit this condition dependence to increase understanding of genetic control of phenotypes.

Susceptibility to alcoholism is one example of a complex phenotype that is controlled by both environmental and genetic factors. Alcohol abuse disorders are intricately connected to environmental perturbations such as stress and anxiety. Stress increases vulnerability to alcohol abuse—both initial use/abuse and relapse—in alcoholics [9], and alcohol has a notable stress-dampening (anxiolytic) effect [10]. Additionally, vulnerability to alcoholism and responses to stress vary widely among individuals, in part because reactions to both alcohol [11] and stress [12] are influenced by genetic factors. Rodent models offer a way to tightly control both environmental and genetic sources of variation and are widely used to study the genetic basis of differences in response to stress and ethanol. For example, the LXS [13], [14], [15] and BXD [16] families of recombinant inbred (RI) strains of mice have been used to discover several quantitative trait loci (QTLs) and candidate genes that control ethanol responses [17], [18], [19], [20].

We attempt to determine how genetic variants and treatment conditions modulate mRNA transcription to produce variation in complex phenotypes [21], [22]. We collected hippocampal gene expression and behavioral phenotype data for LXS and BXD strains under four different treatment conditions: a) SC (saline control), a control group which was only injected with physiologic saline, and not treated with restraint stress, b) EC (ethanol control), a group that was injected with ethanol (1.8 g/kg) but was not otherwise stressed by acute restraint, c) SR (saline and restraint), a group that was stressed acutely by restraint (15 minutes) and then injected with saline, and d) ER (ethanol and restraint), a group that was stressed acutely by restraint (15 minutes) and then injected with ethanol (1.8 g/kg). The hippocampus is well known to be both involved with stress response [23] and sensitive to ethanol [24]. Furthermore, many studies have identified hippocampal gene expression differences involved in responses to stress and ethanol [24], [25], [26], [27], [28], [29], [30], [31], [32], [33], [34], [35]. We investigated treatment-dependent variations in both gene expression and anxiety-related behavioral phenotypes using two sets of recombinant inbred (RI) mice, the LXS and BXD families, allowing for comparisons across 8 different combinations of treatment and population.

The main goal of our study was to determine the genetic and transcriptional basis of natural variation of complex phenotypes associated with responses to stress and ethanol. We identified quantitative trait loci (QTLs) that are responsible for variation in phenotypes and gene expression and determined whether these QTLs were conserved across treatments and populations. To investigate treatment-dependent responses, such as ethanol activation and ethanol-induced anxiolysis, we introduced QTL activity patterns, which show how QTL activities are changed and/or conserved across treatments as well as across the LXS and BXD families. The QTL activity patterns identify treatment-specific QTLs (i.e., QTLs that only had a significant effect under a single treatment) and QTLs that were conserved across multiple treatments. We used similarities in the QTL activity patterns for behavioral phenotypes and gene expression to identify candidate genes that may be responsible for variation in responses to stress and ethanol across strains and treatment groups.

## Results

### The behavioral QTLs are treatment- and population-dependent

Phenotypes measuring the activity count (ACTCNT, beam breaks per second) and percentage of time spent in open quadrants (OPEN) of an elevated zero-maze were used to investigate the effects of ethanol and stress on behavior of the RI strains. The behavior of mice was measured for 0–5, 5–10, and 0–10 (total) minutes after being placed in the zero-maze. Both phenotypes showed large variations across both LXS and BXD strains and treatment groups (Figures S1 and S2 and Table S1). For each treatment group and time period, ACTCNT, which is related to both basal and ethanol-associated locomotor activation, had at least a two-fold difference between the maximum and minimum measurements across strains for both BXD and LXS populations. The anxiety-associated phenotype, OPEN, also varied across strains according to treatment group and time period, with at least a 1.5 fold difference.

**Figure 1:**
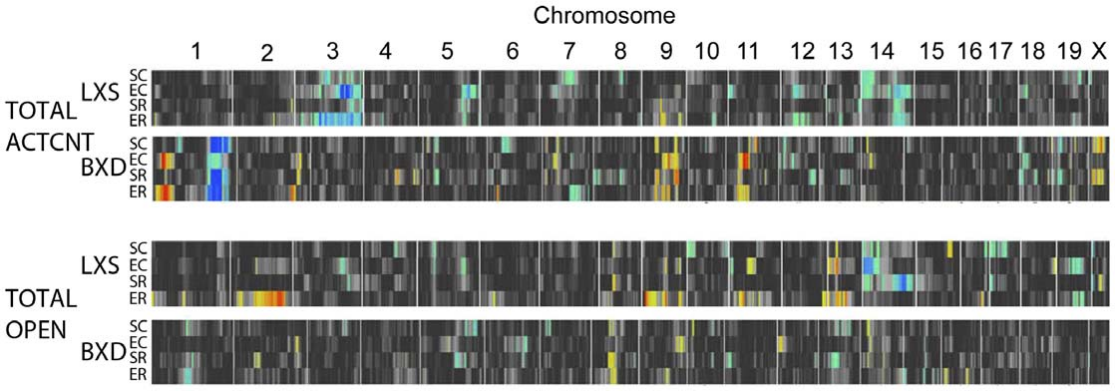
QTL heatmap for behavioral phenotypes. QTL mapping results for two anxiety-related phenotypes, total activity count (TOTAL ACTCNT) and total time spent in open quadrants (TOTAL OPEN), during the time spent in an elevated zero-maze. For each phenotype, each row, from top to bottom, represents the treatment groups SC, EC, SR, ER, respectively. Red indicates a positive correlation between the phenotype and the ILS or DBA/2J genotype at the marker, while blue indicates positive correlation between the phenotype and the ISS or C57BL/6J genotype at the marker. ILS and ISS are the parental strains of the LXS panel. C57BL/6J and DBA/2J are the parental strains of the BXD panel.

QTL mapping was performed to identify genetic loci influencing the locomotor and anxiety-related phenotypes. Treatment with stress and/or ethanol influences the associations between genetic variations and phenotypes across the LXS and BXD strains (Figure 1 and Table S2). In the BXD population, two significant QTLs (genome-wide *p*<0.05) were found on chromosome (Chr) 1 near 25±5 and 158±10 Mb for the ACTCNT phenotype. In the LXS population, significant QTLs (genome-wide *p*<0.05) were found on Chr 3 near 136±5 Mb for the ACTCNT phenotype and on Chr 14 near 9±10 Mb for the OPEN phenotype. These QTLs were not conserved across populations and were highly dependent on treatment with stress and/or ethanol. In the LXS population, the two significant QTLs regulated behavior in the EC group, which included treatment with ethanol but not restraint stress. The Chr 3 QTL, associated with activity count, is located near several previously identified QTLs that have been linked to ethanol-induced locomotion [18], [36] and ethanol-induced loss of righting response [17]. We have previously investigated the Chr 3 QTL which encompasses a cluster of alcohol dehydrogenase genes. *Adh5* was identified as a likely candidate causative gene at this locus [37]. We also observed treatment-dependent behavioral QTLs in the BXD panel, as ACTCNT was associated with two treatment-dependent QTLs on Chr 1. For the SC, SR, and ER groups, a significant QTL was located at 160 Mb, near a hotspot on Chr 1 from 172–178 Mb that controls a diverse assortment of neural and behavioral phenotypes [35]. Similar to the LXS panel, QTL mapping results for the EC group in the BXD panel differed from the other groups, as the significant QTL for activity count was located near 25 Mb on Chr 1 after treatment with ethanol alone (EC). Additionally, the influence of the distal (160 Mb) QTL of Chr 1 on activity count was reduced in the EC treatment.

### Expression QTL classification and activity patterns

**Figure 2:**
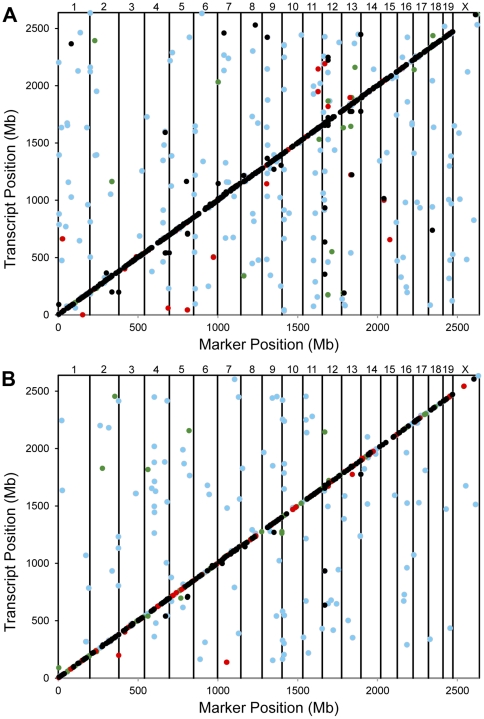
Expression QTL mapping. Significant (FDR <5%) eQTLs are shown for (a) LXS and (a) BXD RI panels. The x- and y-axes show the physical position of the marker and transcript, respectively. The vertical lines indicate the separation of the chromosomes, which are denoted at the top of the figure. Expression QTLs that are conserved in 3, 2, and 1 other treatment conditions are indicated by black, red, and green dots, respectively. Blue dots indicate treatment specific eQTLs.

In order to study treatment dependent genetic regulation of gene expression, we measured gene expression in the hippocampus for each treatment group in the two reference populations. Samples used for obtaining gene expression data were collected 4 hours after treatment, providing a spectrum of early, intermediate, and late gene expression responses to treatment [38]; however, the use of this time point may have missed the influence of treatment on some immediate-early and early response genes. We then mapped expression QTLs (eQTLs) for all treatments and populations. Permutation tests yielded estimates that a LRS (Likelihood Ratio Statistics, LRS = LOD×4.61) threshold of 24 for the LXS panel and 26 for the BXD panel would result in a false discovery rate (FDR) of approximately 5%. Therefore, all QTLs with LRS values greater than the corresponding threshold values (24 and 26, respectively) were selected as significant for each population and treatment. 1923 and 1026 significant eQTLs were found for the LXS and BXD panels, respectively (Figure 2 and Table S3). The eQTL were separated into three groups: *cis*-eQTLs, in which the physical location of the gene and marker were located within 5 Mb, syntenic *trans*-eQTLs in which gene and marker were located on the same chromosome but not within 5 Mb, and non-syntenic *trans*-eQTLs, in which the gene and marker were located on different chromosomes.

**Figure 3:**
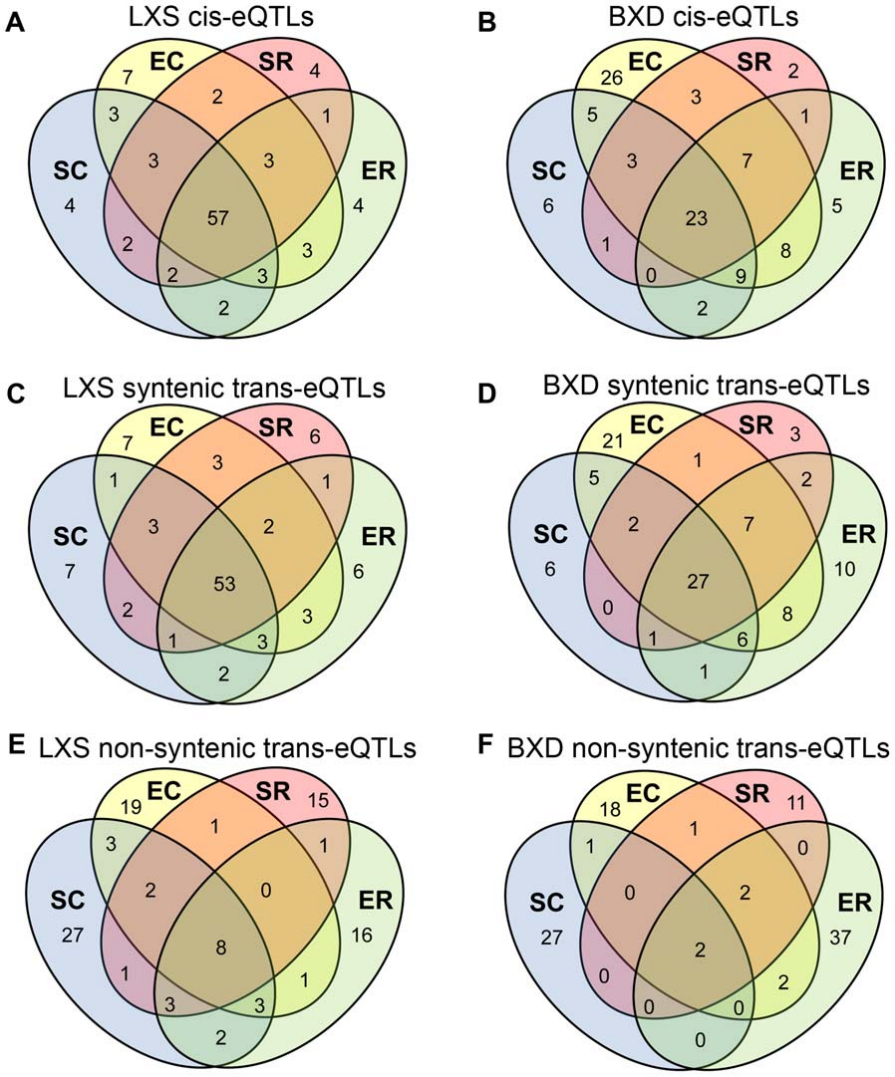
Treatment dependence of eQTL activity. The figure shows the percentage of *cis*-eQTLs [(a) LXS and (b) BXD], syntenic *trans*-eQTLs [(c) LXS and (d) BXD], and non-syntenic *trans*-eQTLs [(e) LXS and (f) BXD] that belong to each of the 15 possible QTL activity patterns. eQTLs are conserved if the LRS >24 for the LXS panel and 26 for the BXD panel for more than one treatment.

The eQTLs were also classified by their QTL activity patterns, which show how the eQTLs were conserved across the various treatment conditions. For each significant eQTL in any given treatment condition, we determined if the eQTL was conserved across other treatments. Based on the conservation of the eQTL across treatments, we then classified the eQTLs into 1 of 15 possible QTL activity patterns (Figure 3).

### Most non-syntenic *trans*-eQTLs are treatment-specific

Approximately 77% and 93% of non-syntenic *trans*-eQTLs were unique to a single treatment in the LXS and BXD panels, respectively (Figure 3 E and F), a result that may be due to *trans*-eQTLs being more sensitive to treatment with stress and/or ethanol. A lower level of conservation of *trans*-eQTLs in comparison to *cis*-eQTLs has been observed in the genetic regulation of stem cells at different stages of development [7] and in human heart cells [8] and yeast cells [6] under different *in vitro* conditions. *Trans*-eQTLs were also found to have a much lower amount of replication across tissues and sexes than *cis*-eQTLs in genetic crosses of the C3H/HeJ and C57BL/6J strains [39].

### *cis*-eQTLs and syntenic *trans*-eQTLs are more conserved across treatments

Conservation of *cis*-eQTLs and syntenic *trans*-eQTLs was much more common (Figure 3 A, B and C, D). For the LXS population, 57% of the *cis*-eQTLs were conserved in all treatment groups, while 23% of the *cis*-eQTLs were conserved in all treatment groups in the BXD panel. While many *cis*-eQTLs were conserved across all treatments and were, therefore, not influenced by treatment with stress or ethanol, we did observe *cis*-eQTLs that were only active under specific treatments. The *cis*-eQTLs with treatment specific activity patterns indicate genes whose expression was influenced by interactions between genetics and treatment with stress and/or ethanol. In both reference populations, the treatment that resulted in the greatest number of *cis*-eQTLs unique to a single treatment group (i.e., eQTLs that were only found in a single treatment group) was the ethanol only (EC) treatment. This result mirrors the patterns found in the anxiety-related phenotypes, where the behavioral QTL mapping under the EC treatment varied from the other treatments.

### Conservation of eQTLs between LXS and BXD populations

We examined conservation of eQTLs between different populations under multiple experimental treatments using two different requirements. First, we determined the eQTLs present under any treatment of one population that were also present under any treatment in the second population (Table S4). 34% of the eQTLs in the BXD panel met this requirement and were conserved in the LXS panel, while 16% of the eQTLs in the LXS panel were conserved in the BXD panel. This increased conservation of BXD eQTLs in the LXS population was likely due to the fact that we observed almost twice as many significant eQTLs in the LXS panel than in the BXD panel, and it was therefore more likely that a given combination of a probe and locus would be significantly associated in the LXS population.

**Figure 4:**
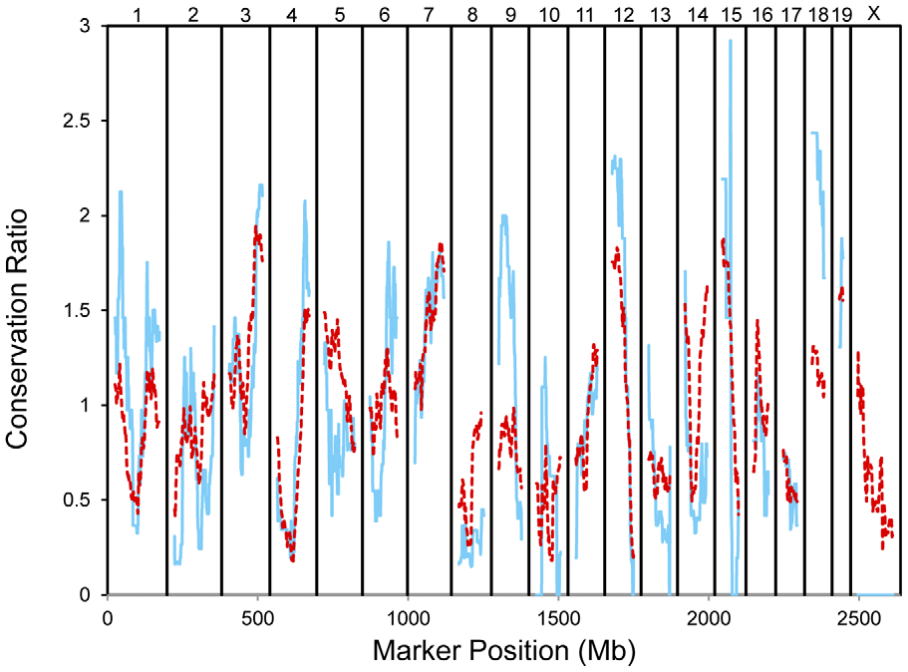
Conservation of eQTLs and SNPs between BXD and LXS populations. The conservation ratio for eQTLs (solid blue line) and SNPs (dashed red line) is shown as a function of genome position. The conservation ratio was calculated from the ratio of the number of features (eQTLs or SNPs) in the BXD population in a sliding window of 50 Mb that were conserved in the LXS population to the expected number of conserved features, given the number of features in the window and the fraction of conserved features across the entire genome. Most locations with a high amount of conservation of eQTLs also have a high amount of conservation of SNPs.

Comparisons of the eQTLs between the two populations also revealed regions where most eQTLs are not conserved and regions where most eQTLs are conserved. For example, while only 6 of the 49 BXD eQTLs located from 0 to 131 Mb on Chr 2 are conserved in the LXS panel, 6 of the 7 BXD eQTLs at a locus near 139 Mb on Chr 2 are conserved in the LXS panel. These patches of non-conserved and conserved eQTLs occur across the entire genome. To quantify this observation, we calculated the conservation ratio, the ratio of the number of eQTLs in the BXD population that were conserved in the LXS population over a sliding window of width 50 Mb across the genome to the number of expected conserved eQTLs in that window given the number of eQTLs in the window and the overall fraction of eQTLs that were conserved across the entire genome (Figure 4). Several locations, such as the distal ends of Chrs 3 and 4 and the proximal end of Chr 12, contain a higher than average number of conserved eQTLs. To examine the genetic basis of this phenomenon, we collected 78881 SNPs in the parental strains of the LXS and BXD populations from the Broad2 haplotype resource [40] available in the Mouse Phenome Database [41]. We determined the SNPs that were conserved across populations and how the conservation ratio of SNPs varied across the genome, using the same method as discussed above for eQTLs. Similar to the conservation of eQTLs, the conservation of SNPs between the LXS and BXD strains is variable across the genome. Additionally, genomic locations with high levels of eQTL conservation often have high levels of SNP conservation, and the conservation ratios for eQTLs and SNPs were highly correlated (Pearson's r = 0.74).

**Figure 5:**
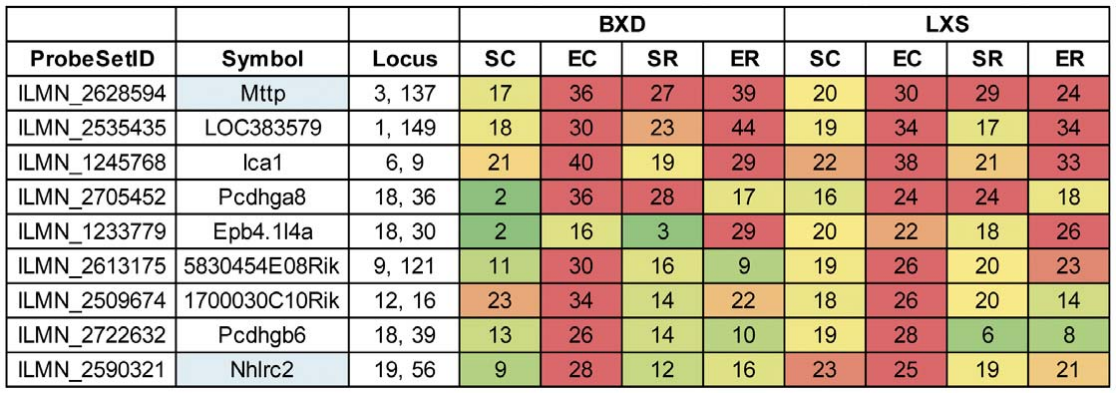
eQTLs with conserved QTL activity patterns. The figure shows the maximum likelihood ratio statistic (LRS) for association between selected traits and loci for each experimental treatment and RI population. The LRS values of eQTLs are color-coded from green (no association, LRS = 0) to red (significant association, LRS = 24 for LXS and LRS = 26 for BXD, FDR = 5%), indicating the strength of the association between the locus and the trait. Genes that have been previously associated with ethanol have a blue background.

Second, we determined the eQTLs that were present in both populations that also had identical eQTL activity patterns in both populations (Table S4). A small fraction of the eQTLs (9% of BXD eQTLs and 5% of the LXS eQTLs) were conserved in this manner, and the majority of these eQTLs were conserved across all treatments in both populations. Only 9 eQTLs, which were all *cis*-eQTLs, were conserved across populations with treatment dependent QTL activity patterns (Figure 5). The majority of these eQTLs were not present under the control, SC treatment, but were present after some combination of treatment with stress and/or ethanol, indicating that these eQTLs were activated only after stress/ethanol treatment. Three genes with conserved eQTL activity patterns, *Pcdhga8*, *Pcdhgb6*, and *Epb4.1l4a*, are all located on Chr 18 near 135 Mb but display different eQTL activity patterns. The eQTLs for *Pcdhg6* and *Epb4.1l4a* were significant only after the EC and ER treatments, respectively, while *Pcdhga8* had a significant eQTL after both EC and SR treatments. Additionally, *Mttp*, another gene with a conserved eQTL activity pattern, is controlled by the same locus (Chr 3 near 135 Mb) as the behavioral QTL regulating activity count in LXS strains and has been previously linked to ethanol abuse in humans [42].

### Comparison of behavioral and expression QTL activity patterns

Behavioral QTLs that explain variation in anxiety-related phenotypes were dependent on treatment with stress and/or ethanol as well as the RI panel used. In order to reveal the genes that confer variation in genotype to treatment and population dependent variation in phenotypes, we created activity patterns for the eQTLs that colocalized with the three significant behavioral QTLs that were activated by treatment with ethanol. We hypothesized that the variation in behavioral phenotypes and their dependence on treatment conditions and reference population could potentially be explained by finding genes with eQTLs that were colocalized with the phenotype QTLs and, also, had similar treatment and population dependent QTL activity patterns. We first identified three key features of the behavioral QTL activity patterns: 1) the QTL was not associated with the trait in the control SC group, 2) the QTL had a significant association with the trait in the ethanol-treated EC group, and 3) the QTL was not conserved across LXS and BXD populations. For each of the behavioral QTL regions, genes with eQTL activity patterns that met these three conditions were selected.

**Figure 6:**
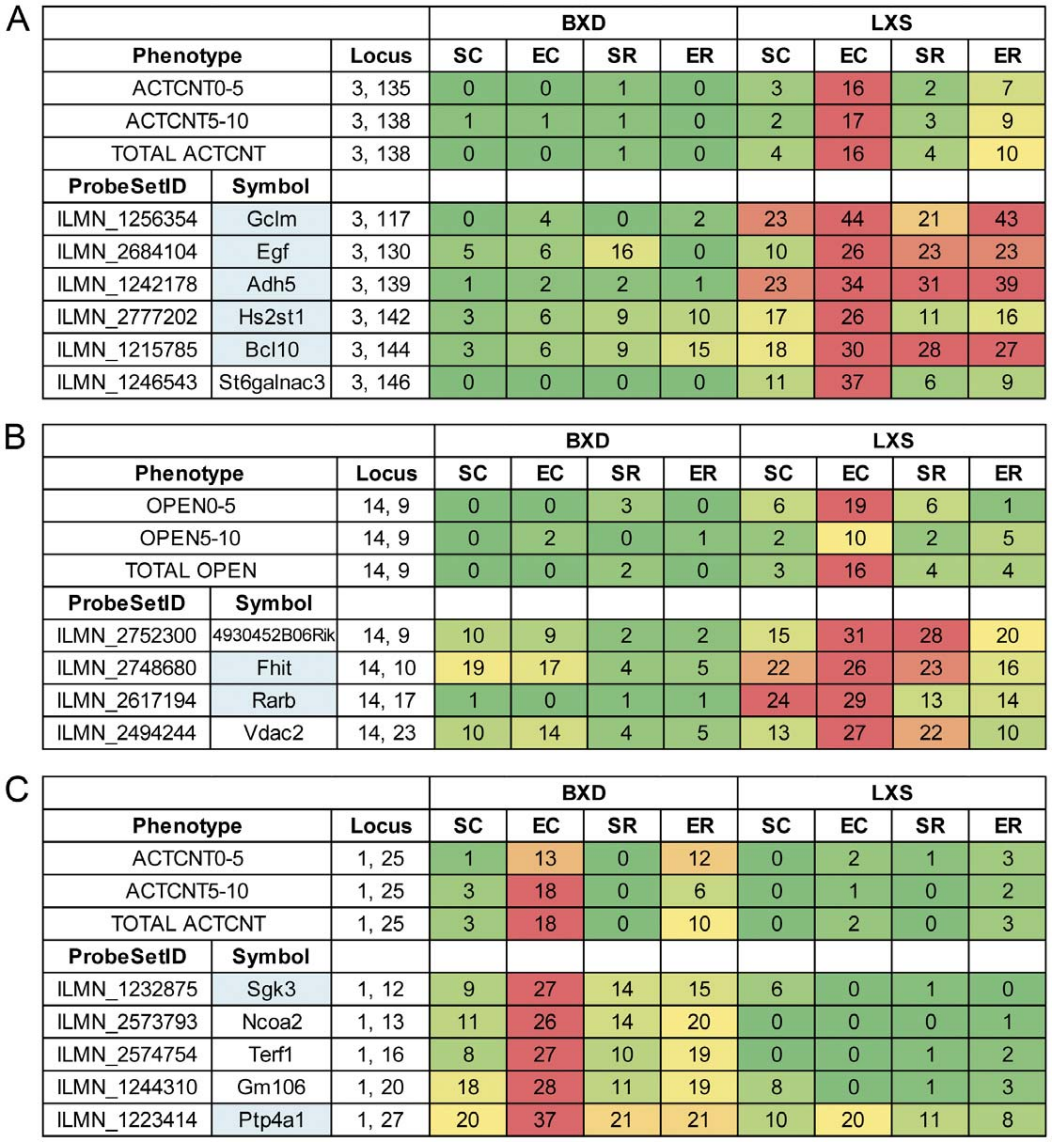
QTL activity patterns. The figure shows the maximum likelihood ratio statistic (LRS) for association between selected traits for each experimental treatment near 136 Mb on Chr 3 (a), 9 Mb on Chr 14 (b), and 25 Mb on Chr 1 (c), the locations of the three significant behavioral QTLs. All gene expression transcripts that had an eQTL activity pattern similar to the phenotype QTL activity pattern are shown. The LRS values of eQTLs are color-coded from green (no association, LRS = 0) to red (significant association, LRS = 24 for LXS and LRS = 26 for BXD, FDR = 5%), indicating the strength of the association between the locus and the trait. Genes that have been previously associated with ethanol have a blue background.

We identified six genes with eQTLs with similar locations and activity patterns as the behavioral QTL regulating activity count on Chr 3 near 136 Mb that was significant only in the LXS panel after the EC treatment (Figure 6A). Interestingly, *Adh5*, alcohol dehydrogenase 5, was one of the selected genes. The reduced dependence of the expression of *Adh5* on genotypic variation at Chr 3 in the BXD panel may, to a large extent, explain why activity count was not associated with the locus on Chr 3, suggesting that the BXD panel may lack the genetic polymorphism that causes variation in this phenotype. Several of the other six genes have been previously identified as being ethanol-associated according to the Ethanol Related Gene Resource (ERGR) [43] and Gene Ontology annotations. For example, *Egf* was identified as a possible ethanol-related gene in humans [42] that is involved in the MAPK signaling pathway, which has been linked to stress and alcohol, and *Hs2st1* has been associated with ethanol preference in mice [44].

The second behavioral QTL in the LXS panel, which was located on Chr 14 near 9 Mb and controlled the time spent in open quadrants, had a similar treatment and population dependence as the Chr 3 behavioral QTL. Four genes had similar LXS eQTL activity patterns (Figure 6B), and two of these, *Vdac2* [45] and *Rarb* [46], have been previously associated with response to alcohol. We performed a similar procedure for the BXD behavioral QTL on Chr 1 near 25 Mb that was active only after the EC treatment. Five genes with similar BXD eQTL activity patterns were found (Figure 6C), and two of these have been previously associated with alcohol response. *Sgk3* and *Ptp4a1* have both been previously shown to be differentially expressed in mouse brain after treatment with ethanol [38], [44], [47].

**Figure 7:**
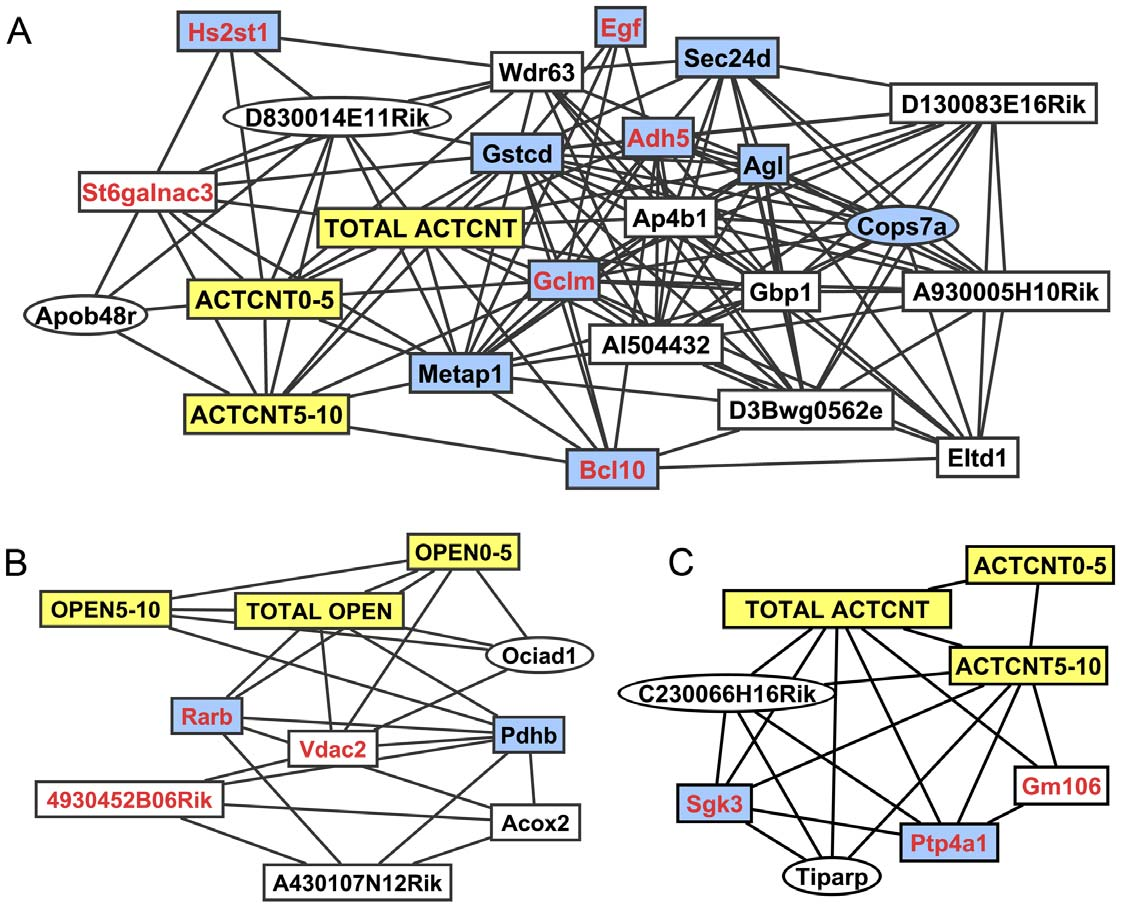
QTL activity pattern correlation network. The correlation networks were created from the Pearson correlation between QTL activity patterns of behavioral phenotype and gene expression transcripts at three locations with treatment-dependent behavioral phenotype QTLs: Chr 3 from 126 to 142 Mb (a), which was associated with the ACTCNT phenotype in the LXS panel, Chr 14 from 0 to 24 Mb (b), which was associated with the OPEN phenotype in the LXS panel, and Chr 1 from 22 to 32 Mb (c), which was associated with ACTCNT in the BXD. Traits are connected in the network if the Pearson correlation coefficient of the QTL activity pattern is greater than 0.8. Nodes with yellow and blue backgrounds denote phenotypes and genes that have been previously associated with ethanol, respectively. Genes that had similar QTL activity patterns as the phenotypes (Figure 6) are in red text. Elliptical nodes identify genes that are located on different chromosomes than the behavioral QTLs.

To further investigate how QTL activity patterns can be used to identify genes that influence phenotypes, we created correlation networks to find gene expression traits with QTL activity patterns similar to the QTL activity patterns of behavioral phenotypes (Figure 7). The QTL activity patterns for gene expression transcripts similar to the three ethanol-related behavioral QTLs (Chr 1 near 25 Mb, Chr 3 near 136 Mb, and Chr 14 near 9 Mb) were identified. The QTL activity pattern correlation networks included several of the genes, including *Adh5*, *Bcl10*, *Vdac2*, *Rarb*, *Sgk3*, and *Ptp4a1* that were identified as potential candidate genes in the previous procedure (Figure 6). Several of the new genes included the correlation networks, including *Pdhb*, *Metap1*, *Gstcd*, and *Agl* have been previously associated with ethanol in the ERGR [43].

## Discussion

We used the LXS and BXD panels of recombinant inbred mice to investigate the genetic basis of variation in response to stress, ethanol, and their combination and how these responses are altered and/or conserved across these populations. The QTLs that controlled two anxiety-related behavioral phenotypes (ACTCNT and OPEN) were highly dependent on treatment and population, and we introduced the concept of QTL activity patterns which show how the strength of the association between a genomic location and a trait varies across different experimental conditions. We then used similarities between behavioral QTL activity patterns and eQTL activity patterns to identify genes that may be responsible for conferring genomic and experimental variation in phenotypes (Figures 6 and 7). We identified several genes by this method, including *Adh5*, *Hs2st1*, *Vdac2*, *Rarb*, *Sgk3* and *Ptp4a1*, which have been previously associated with responses to ethanol, and novel genes, including *Ncoa2*, *St6galnac3*, *Gm106*, and *Terf1*, which, to our knowledge, have not been directly linked with ethanol. *Adh5*, a candidate gene for the ethanol-dependent QTL for activity count on the distal end of Chr 3, is of particular interest, as the gene is part of a cluster of alcohol dehydrogenase (ADH) genes on the distal end of Chr 3. Genetic variations in this ADH cluster, which is located on human chromosome 4, have been strongly associated with alcoholism in several human studies [48], but there have yet to be associations between this gene cluster and ethanol response QTLs in mouse [49]. *Vdac2*, which encodes an anion channel in the outer membrane of mitochondria, has been shown to function in the hippocampus [50] and has been associated with neuroactive steroids and the GABA-A receptor [51]. *Ncoa2*, which functions as a steroid receptor coactivator in the hippocampus [52], may be involved in the postnatal stress hypo-responsive period in mice [53], connecting this gene with glucocorticoid receptors linking stress and addiction [54]. *Sgk3*, a member of the serum- and glucocoriticoid-inducible kinase family, is highly expressed in the brain and has been shown to regulated hippocampal abundance of the GluR1 subunit of the AMPA-type glutamate receptor [55]. *Ptp4a1* plays a regulatory role in a variety of cellular processes and was recently identified as a potential gene harboring a causal locus in genome-wide association studies in several human populations [56].

These QTL activity patterns may also be applied to identify genes responsible for previously identified behavioral QTLs. The LXS RI strains and their parental strains have been used to study the genetic basis of several alcohol-associated phenotypes, including ethanol-induced loss of righting due to ethanol (LORE). Four well-characterized behavioral QTLs (*Lores1*, *2*, *4*, and *5*) explaining variation in LORE have been identified [57], but the genes that underlie these QTLs have yet to be determined. While the specific ethanol treatment protocols and phenotypes studied differed between the LORE studies and this work, the expression of many genes that underlie the LORE QTLs may be susceptible to treatment with ethanol in general, and we, therefore, used eQTL activity patterns to select genes with ethanol-activated QTLs in the LORE QTL regions (Table S5). The analysis of QTL activity patterns in *Lore4*, which is located on Chr 11 from 79–106 Mb, in particular, revealed several interesting genes that may explain the variation in ethanol response in this region. *Ccl3* has a *cis*-eQTL in *Lore4* that is significant only under EC treatment. *Ccl3*, chemokine (C-C motif) ligand 3, has been identified as a candidate gene associated with the LORE QTL, influencing alcohol preference and alcohol consumption [58]. The *Ccl3* eQTL is not conserved in the BXD population. Other genes which contain an ethanol activated eQTL in *Lore4* include *Grb7*, *Stxbp4*, *Hexim2*, and *Smarce1*, which has been previously associated with ethanol metabolism [59]. Several genes with eQTLs located near the other LORE QTLs, including *Rassf2* and *Pcsk2* which have eQTLs in *Lore2* and *Ctrhc1*, *Mfsd1*, and *Mast2*, which have eQTLs in *Lore5*, have been previously associated with ethanol [43]. Additionally, the expression of several genes involved in molecular pathways that are known to link stress with addiction and substance abuse were found to be regulated by *cis*-eQTLs in this study (Table S3), including several genes associated with the cAMP response element-binding (CREB) protein, a transcription factor involved in responses to stress, drug exposure, and their interacting effects [60]. A *cis*-eQTL regulates expression of CREB5 in BXD mice after SC, EC, and ER treatments, while CAMK4, which has been shown to phosphorylate and activate CREB [61], has a *cis*-eQTL that is conserved across treatments and families. The conservation of eQTLs across populations was also investigated. We observed that, at some genomic locations, most eQTLs were conserved between the LXS and BXD populations, while conservation was rare at other locations. These patches of conserved eQTLs could be useful in narrowing down the candidate genetic polymorphisms influencing gene expression because only the common genetic polymorphisms in both populations could be responsible for the QTL effect.

While many eQTLs were conserved across populations when all possible treatments were considered, the conservation of entire eQTL activity patterns was relatively rare. This result may have been due to using a rather strict criterion for determining if the eQTL activity pattern was conserved, as we required that conserved eQTLs met the cutoff for significance used to select significant eQTLs (i.e., LRS >24 for LXS and LRS >26 for BXD). In many cases, eQTLs that were potentially conserved across treatments or populations were suggestive but did not meet this threshold for significance. However, the assignment of a QTL to a particular class of activity patterns is not required in many cases, and other methods, such as calculating the correlation between QTL activity patterns, are useful for investigating how treatment conditions influence QTLs. For example, we used correlations between the activity patterns of behavioral phenotype QTLs and eQTLs to identify genes that may influence the behavioral phenotypes (Figure 7).

## Materials and Methods

### Ethics Statement

All procedures involving animals were approved by the Animal Care and Use review boards of The University of Tennessee Health Science Center and The University of Memphis. The protocol numbers are 680 and 0609, respectively.

### Recombinant inbred mice

The LXS strains were obtained from Dr. Beth Bennett and colleagues at the University of Colorado, Boulder. All animals were raised at the University of Colorado or the University of Memphis in SPF facilities and were between 60 and 74 days of age. The same 31 LXS strains were present in each treatment group for both phenotype and gene expression studies. The BXD strains were raised at the University of Tennessee Health Science Center and were between 60 and 95 days of age. 29, 36, 29, and 30 BXD strains were used in the gene expression studies under SC, EC, SR, and ER treatments, respectively. Behavioral phenotype data was collected for 71, 70, 70, and 69 BXD strains under SC, EC, SR, and ER treatments, respectively. For behavioral data, the average value for the strain (typically from 6 males and 6 females) was used in further analysis, while the expression data was obtained for at least 2 animals per strain and condition in most cases. Further details are available on the GeneNetwork (www.genenetwork.org).

### Behavioral phenotypes

The phenotypes were measured for LXS and BXD mice for 4 treatment groups: SC, EC, SR, and ER. In the SC treatment, mice were injected with a single IP injection of 0.018 ml of 0.9% saline per g of mouse weight, but were not subjected to any additional stress other than that associated with handling and the injection. In the EC treatment, mice were injected with 1.8 g/kg of ethanol, but had no additional stress. In the SR treatment, mice were subjected to restraint stress by being placed in a conical restraining tube for a period of 15 minutes and were then injected with saline. A 50 ml conical centrifuge tube is used as the restraint stress tube. The tip of the conical end of the tube is cut off to provide aeration. The mouse is placed, head first, into the tube and the end cap screwed on. A hole drilled in the end cap accommodates the length of the mouse's tail. The tube is placed between wedges to prevent the tube from rolling during the restraint stress period. In the ER group, the mice were subjected to restraint stress and were injected with ethanol. Five minutes after injection with saline or ethanol, the mice were placed in an elevated zero-maze, which is commonly used to measure the anxiety level and activity of mice, for 10 minutes and their behavior was monitored. The behavior of the mice was measured using two phenotypes over three periods of time. Activity count (ACTCNT) was calculated as the average number of beam breaks of infrared sensors in closed quadrants per second over minutes 0–5 (ACTCNT0–5), minutes 5–10 (ACTCNT5–10), and minutes 0–10 (TOTAL ACTCNT) of the time spent in the maze. The second phenotype was the percentage of time spent in open quadrants (OPEN) over minutes 0–5 (OPEN0–5), minutes 5–10 (OPEN5–10), and minutes 0–10 (TOTAL OPEN). Behavioral testing was carried out at the University of Memphis.

### RNA isolation and microarray experiments

The mice were treated with stress and/or alcohol, as discussed previously. Mice were sacrificed by cervical dislocation 4 hours after injection with saline or ethanol; thus animals were typically sacrificed between 1:30 and 3 p.m. Brains were quickly removed and stored in RNAlater. Brains were frozen at −80°C until being transferred to the University of Tennessee Health Science Center. Whole brain dissections were performed at the University of Tennessee Health Science Center. Hippocampal samples were close to complete but were likely to include variable amounts of fimbira and choroid plexus and parts of the subiculum. The bilateral hippocampus tissue from one naïve adult mouse was used to generate RNA samples. RNA purity was evaluated using the 260/280 nm absorbance ratio, and values had to be greater than 1.8 to pass quality control. The majority of samples had values between 1.9 and 2.1. RNA integrity was assessed using the Agilent Bioanalyzer 2100. The standard Eberwine T7 polymerase method was used to catalyze the synthesis of cDNA template from polyA-tailed RNA using the Ambion/Illumina TotalPrep RNA amplification kit. The biotin labeled cRNA was then evaluated using both the 260/280 ratio (values of 2.0–2.3 are acceptable) using a NanoDrop ND-1000. Those samples that passed quality control steps were immediately used on Illumina Sentrix Mouse 6.1 Bead arrays. The slides were hybridized and washed following standard Illumina protocols. All samples were scanned on a single Illumina Beadstation. The data set was processed and normalized using the standard Rank Invariant method developed by Illumina and described in their BeadStation Studio documentation.

### Genotype and microarray annotation

2659 SNPs and microsatellites typed across LXS strains and 3796 SNPs and microsatellites typed across BXD strains were used in QTL mapping. The genotypes and their genome locations used are freely available on the GeneNetwork at http://genenetwork.org/dbdoc/LXSGeno.html and http://genenetwork.org/dbdoc/BXDGeno.html. An annotation file available on the GeneNetwork Data Sharing Zone (http://www.genenetwork.org/share/annotations/) was used to determine the genes and genome locations associated with the 46,643 unique probe sequences in the Illumina Mouse 6.1 array.

### QTL mapping

QTL mapping of behavioral phenotype data was performed using the batch submission feature of the GeneNetwork. An empirical genome-wide p-value for association between the phenotype and each marker was determined from 1000 permutations of the data. QTL mapping of gene expression data was performed using QTL Reaper for each data set independently. The likelihood ratio score (LRS) for each combination of probe set and marker was calculated with QTL Reaper. For each probe set, QTLs were defined by selecting the marker on each chromosome with the maximum LRS. 1000 permutations of the strain labels were performed to determine false discovery rates (FDR) for several LRS thresholds [62]. LRS thresholds of 24 and 26 resulted in a FDR of ∼5% for each of the four treatment groups for the LXS panel and BXD panel, respectively. For eQTLs identified in the BXD panel, we used the recently compiled sequence of the DBA/2J strain [63] to identify probes that contained polymorphisms between the BXD parental strains. We removed all *cis*-eQTLs that were identified using probes containing SNPs that had higher expression for strains with the B6 allele and were conserved across all treatments. As the parental strains of the LXS panel have not been fully sequenced, we were unable to perform a similar filtering for the LXS eQTLs. QTL mapping results for both behavioral phenotypes and expression traits across the entire genome can be viewed on the GeneNetwork.

### QTL activity patterns

Conservation of eQTLs across treatment groups for each panel was determined by first selecting all probe and marker combinations that resulted in significant eQTLs. Then, the LRS value for association between the marker and probe was calculated under all treatments. If the LRS value exceeded 24 for the LXS panel and 26 for the BXD panel under a given treatment, the eQTL was considered to be conserved in that treatment.

Conservation of eQTLs across LXS and BXD populations was determined by first determining the BXD marker nearest each LXS marker and the LXS marker nearest each BXD marker. Then, the probe set and corresponding BXD marker was selected for the probe set and LXS marker of each significant eQTL in the LXS data. The LRS value for association between the probe set and BXD marker was calculated for each treatment in the BXD data, and the eQTL was conserved under a given treatment if the LRS value was greater than 26. A similar procedure was used to determine conservation of BXD eQTLs in the LXS data, except an LRS value of 24 was used as the cutoff. To test if the possibility of differences between the locations of the nearest markers in the LXS and BXD data had a significant effect on the determination of conservation of eQTLs across populations, we also determined the eQTLs that were conserved for only those eQTLs in which the identical marker was used for both the LXS and BXD populations. While the fraction of conserved eQTLs between LXS and BXD populations with these identical markers were typically higher than what was found when comparing the nearest marker eQTLs, the general trends found for conserved eQTLs were similar (Table S4).

### Correlation networks based on QTL activity pattern

Correlation networks were created from Pearson correlations between LRS values for behavioral QTLs and eQTLs. The behavioral QTLs used were Chr 3 from 126 to 142 Mb, which was associated with ACTCNT in the LXS panel after EC treatment, Chr 14 from 0 to 24 Mb, which was associated with the OPEN phenotype in the LXS panel after EC treatment, and Chr 1 from 22 to 32 Mb, which was associated with ACTCNT in the BXD panel after EC treatment. Next, the LRS values for association between these loci and all gene expression transcripts were calculated for each treatment and population. The gene expression traits were filtered to select traits with LRS >20 (FDR∼25%) for association with the loci after EC treatment in the LXS panel for the Chrs 3 and 14 QTLs and in the BXD panel for the Chr 1 QTL. Additionally, the LRS values for association between the QTLs and the behavioral phenotypes were calculated for all treatments and populations. The Pearson correlation coefficient was calculated for correlations between the LRS values of the selected gene expression transcripts and phenotypes. Transcripts whose QTL activity patterns were associated with the behavioral QTL activity patterns with correlation coefficients >0.7 were included in the QTL activity pattern correlation networks. Connections between traits shown in Figure 7 had eQTL activity patterns with correlation coefficients >0.8.

## Supporting Information

Figure S1 **Variation in activity count in LXS strains as a function of treatment.** Total activity count varies as a function of strain and treatment with stress and ethanol for the LXS population. Data are shown for SC (a), EC (b), SR (c), and ER (d) treatments. The strains were placed along the *x*-axis in order of increasing magnitude of activity count after SC treatment in all panels.
(TIF)

Figure S2 **Variation in activity count in BXD strains as a function of treatment.** Total activity count varies as a function of strain and treatment with stress and ethanol for the BXD population. Data are shown for SC (a), EC (b), SR (c), and ER (d) treatments. The strains were placed along the *x*-axis in order of increasing magnitude of activity count after SC treatment in all panels.
(TIF)

Table S1 Behavioral phenotypes.
(XLS)

Table S2 Behavioral phenotype QTL mapping.
(XLS)

Table S3 Gene expression QTL mapping.
(XLS)

Table S4 Comparison of eQTL activity patterns between BXD and LXS populations.
(XLS)

Table S5 Activity patterns for LXS eQTLs located near LORE behavioral QTLs.
(XLS)
