# Supplementary figures and images for: Treatment- and Population-Dependent Activity Patterns of Behavioral and Expression QTLs
Source: PLoS One. 2012 Feb 16;7(2):e31805. doi: 10.1371/journal.pone.0031805 (PMC3281015; doi:10.1371/journal.pone.0031805)

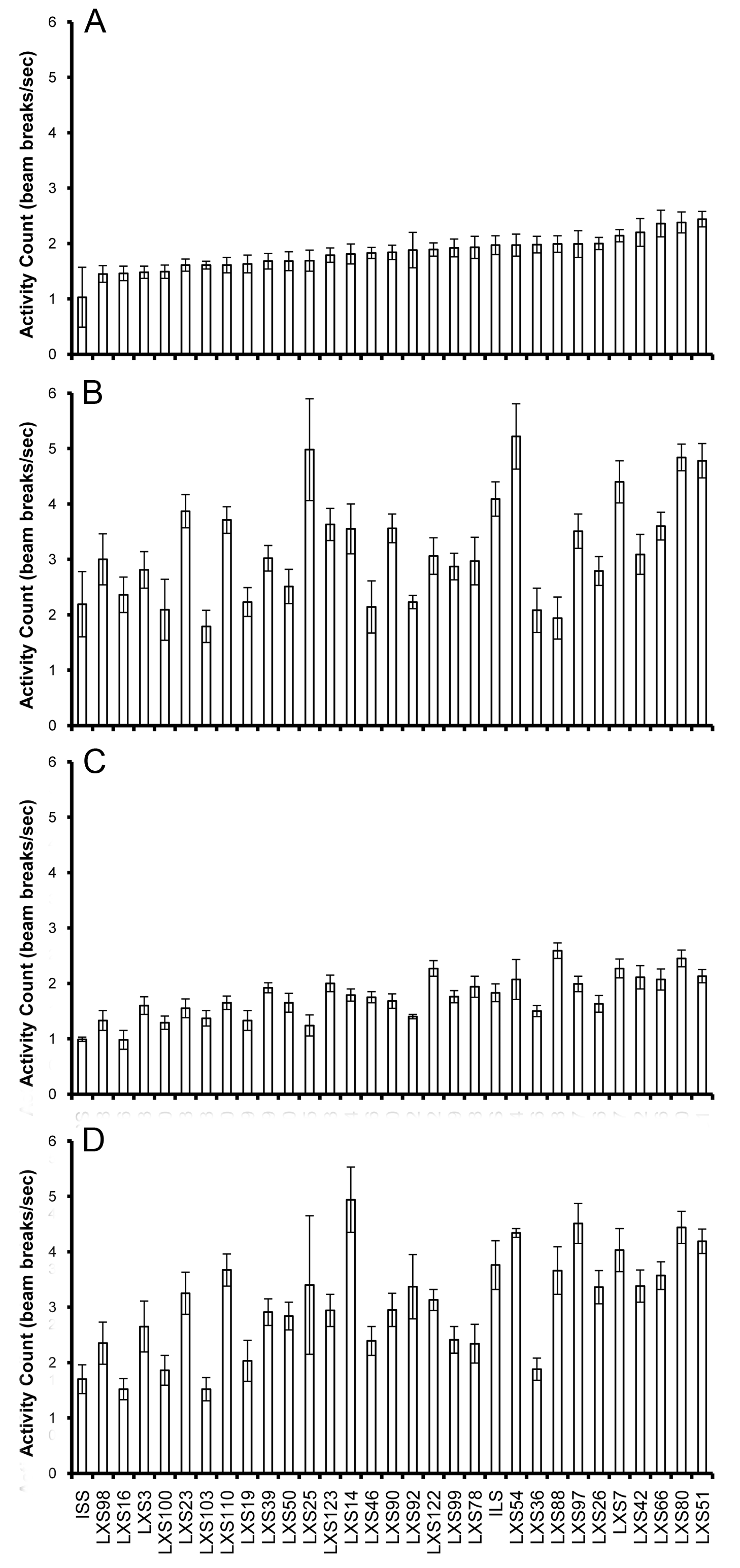

Supplement: Figure S1 — Variation in activity count in LXS strains as a function of treatment. Total activity count varies as a function of strain and treatment with stress and ethanol for the LXS population. Data are shown for SC (a), EC (b), SR (c), and ER (d) treatments. The strains were placed along the x-axis in order of increasing magnitude of activity count after SC treatment in all panels. (TIF) [file pone.0031805.s001.tif]

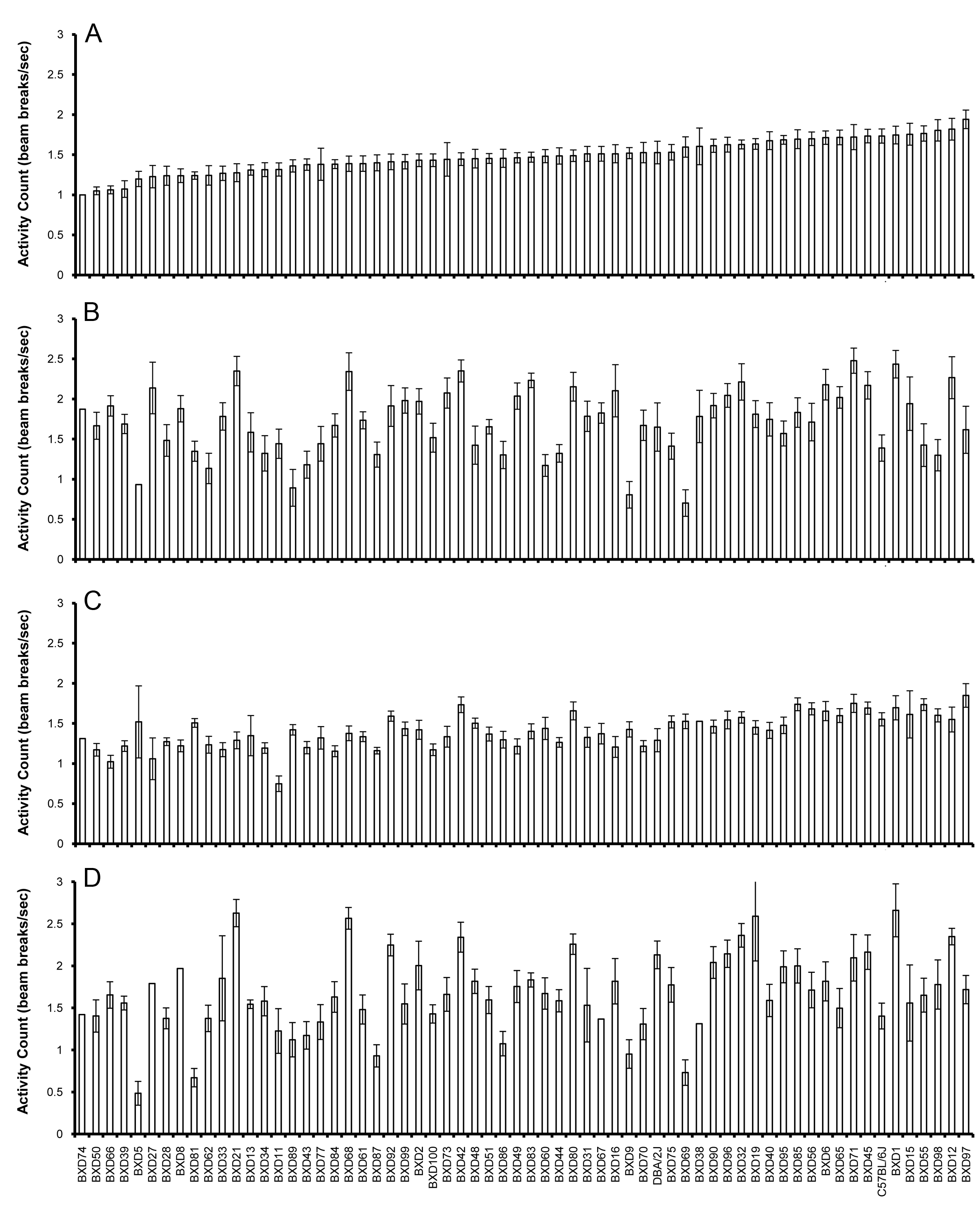

Supplement: Figure S2 — Variation in activity count in BXD strains as a function of treatment. Total activity count varies as a function of strain and treatment with stress and ethanol for the BXD population. Data are shown for SC (a), EC (b), SR (c), and ER (d) treatments. The strains were placed along the x-axis in order of increasing magnitude of activity count after SC treatment in all panels. (TIF) [file pone.0031805.s002.tif]
